# Attributional retraining: Promoting psychological wellbeing in older adults with compromised health

**DOI:** 10.3389/fspor.2022.949501

**Published:** 2022-08-16

**Authors:** Patti C. Parker, Judith G. Chipperfield, Jeremy M. Hamm, Raymond P. Perry, Masha V. Krylova, Loring M. Chuchmach, Steve Hladkyj

**Affiliations:** ^1^Department of Psychology, Thompson Rivers University, Kamloops, BC, Canada; ^2^Department of Psychology, University of Manitoba, Winnipeg, MB, Canada; ^3^Department of Psychology, North Dakota State University, Fargo, ND, United States

**Keywords:** attribution theory, attributional retraining, health challenges, perceived control, helplessness

## Abstract

Older adults make up the largest portion of the population of physically inactive individuals. Health challenges, and psychological barriers (e.g., maladaptive causal attributions), contribute to reduced activity engagement and low perceived control. This pilot study tested an attributional retraining (AR) intervention designed to increase control-related outcomes in a physical activity context for older adults with compromised health. Using a randomized treatment design, we examined treatment effects on a sample of older adults attending a day hospital (*N* = 37, *M*_age_ = 80). We employed ANCOVAs, controlling for age, sex, and morbidity, to assess differences in post-treatment outcomes between AR and No-AR conditions. AR recipients (vs. No-AR) reported lower post-treatment helplessness and more perceived control over their health. Our study offers evidence for AR to increase control-related outcomes and lays the groundwork for further research into supporting older adult populations with compromised health.

## Introduction

Physical inactivity is a significant public and global health concern for all ages and especially for older adults (World Health Organization, 2020). According to the WHO, one in four adults are not meeting recommended standards of physical activity, and those with insufficient levels of activity have a 20–30% increased risk of all-cause mortality than those with sufficient levels. Individuals who are inactive also have a greater risk of cancer, functional limitations, and cognitive decline, and reduced quality of life (Cunningham et al., 2020). Unfortunately, older adults with compromised health may have limited capabilities to engage in physical activity.

Among older adults, individuals with some form of compromised health (e.g., mobility issues, poor self-reported health, autoimmune diseases) often experience challenges that can prevent them from activity engagement (Booth et al., 1997; Rasinaho et al., 2007; Sharif et al., 2018). Compounding this challenge is the way in which older adults cognitively appraise, or make attributions for, their physical inactivity. Blaming inactivity on old age can undermine motivation, reduce adaptive health behaviors, and is linked to higher mortality risk (Parker et al., 2019). Although physical inactivity can be explained by factors such as increasing comorbidities or shrinking social networks, individuals feel in greater control if they focus on other attributions. Our study tests the efficacy of a control-enhancing attributional retraining (AR) intervention to improve quality of life (adaptive control-related outcomes) for older adults with compromised health.

According to attribution theory (Weiner, 1985), attributions used to explain negative events (e.g., achievement failure, physical inactivity) that are personally uncontrollable, rather than controllable, can be maladaptive and harmful for motivation (Chipperfield and Segall, 1996; Perry et al., 2010). Weiner posits causal attributions are classified using three dimensional properties: locus of causality (internal vs. external to the person), stability (stable vs. unstable), and controllability (controlled vs. uncontrolled by the person; Weiner, 1985). Concerning health settings, attributions for poor health outcomes that are internal, stable, and uncontrollable (e.g., “old age”) have negative implications for psychological wellbeing, health behaviors, and survival (Stewart et al., 2012; Parker et al., 2019).

AR interventions that encourage adaptive attributions are shown to benefit motivation-relevant cognitions, emotions, and behavior (Perry et al., 2010; Graham and Taylor, 2021). A rich literature supports the efficacy of AR in achievement settings (Perry and Hamm, 2017). Encouraging people to view their inactivity using internal, controllable, and unstable attributions should have an impact on cognitions. For example, if individuals believe their inactivity is a result of using the wrong strategy (e.g., trying to do too much)—that is, making an unstable and controllable attribution—theoretically they are more likely to believe they can change the strategy (e.g., plan to start slower) and this should enhance their perceived control. The focus of our study was on control-related outcomes known to be critically important for maintaining independence and wellbeing (Robinson and Lachman, 2017).

In a review on perceived control and aging, perceived control is referred to as “one's beliefs about the likelihood that one's actions can bring about desired outcomes” (p. 436; Robinson and Lachman, 2017). Across various life domains, perceived control is important for health and wellbeing, performance, and protecting against challenges in aging (Robinson and Lachman, 2017). For example, perceptions of control have been tied to psychological and affective health and survival (Lachman et al., 2008; Infurna et al., 2013). Chipperfield et al. (2018) proposed that the impact of perceived control may become even more critical in later life as individuals are challenged with greater barriers, such as compromised health and functional independence. Consequently, this highlights the importance for studying perceived control in older adults facing health challenges.

AR has been shown to bolster individuals' perceptions of control in various domains and populations, including achievement in young adults (see Perry and Hamm, 2017); however, this has not been explored in older populations with compromised health. There have been some AR studies conducted on older adults in a physical activity context. AR intervention research that focused on dispelling old age attributions suggested this increased physical activity (Sarkisian et al., 2007). Additionally, another study found AR helped increase walking behavior in older Hispanic and Latino populations (Piedra et al., 2018).

Our pilot study extends this line of research in several ways. First, the premise of our AR intervention is to have older adults consider *various* causes for their inactivity that are within their control to more accurately reflect the complex reality of causal thinking. Second, we examined older adults who had been admitted to a day hospital for a health-related problem. These individuals with various health challenges were admitted to the hospital for a short-term rehabilitation program (e.g., rehabilitating after a health problem such as a stroke or major surgery). Because individuals with compromised health are often at risk of reduced activity, they provided an optimal sample for our consideration of physical inactivity. Third, our study goes beyond past research by examining the role of AR on control-related psychological outcomes (helplessness, perceived control). Improvements in these psychological outcomes should logically follow when people view their inactivity as a result of controllable causes, thus fostering their motivation to become more physically active.

### Study objective

Given the important implications for reducing inactivity in older adults and the efficacy of altering harmful attributions for important outcomes, we sought to provide an AR intervention for older adults at-risk of limited activity. Our main study goal was to test the short-term efficacy of AR on control-related outcomes. Our directional hypotheses were tested using a randomized experimental treatment design for older adults attending a day hospital facing health challenges. AR (vs. No-AR) was hypothesized to decrease helplessness and increase perceived control over health, fitness, and life in general. One-sided tests were used since according to theory and past AR studies, treatment recipients should benefit psychologically from AR (see Perry and Hamm, 2017).

## Method

Our study sample comprised 37 older adults attending a day hospital in Manitoba (aged 67–94, 65% female, 95% English as a first language, 74% attained a high school degree or higher; see Table 1). The hospital staff screened interested patients who were deemed cognitively capable to participate. Objective screening was facilitated by scores on the Mini-Mental State Examination (≥19). Other criteria for participation included: participants planning to return to the hospital on a weekly basis, understanding English, and ability to engage in activity or physical movement. Typical patients at the day hospital are offered a short-term rehabilitation program for undergoing a health-related challenge and attended once or twice a week during the day. Patients were referred to the hospital either from their family doctor, a homecare coordinator, or a geriatric program assessment team. The participants recruited for our study provided written-informed consent and ethics approval was obtained from both the researchers' institution and the health centre of the cooperating day hospital.

**Table 1:** Frequencies and descriptive statistics for demographic and psychological variables.

**Demographic variables^a^**	***M* or frequency (%)**	***SD***	**Range**
Age	80.39	7.70	67–94
Sex	–	–	–
Male	13 (35%)		
Female	24 (65%)		
Marital status		–	–
Never married	2 (5%)		
Married	11 (30%)		
Widowed	17 (46%)		
Separated	2 (5%)		
Divorced	5 (14%)		
Education completed	–	–	–
8^th^ Grade or less	1 (3%)		
Grade 9–11	7 (22%)		
High School	8 (25%)		
Technical/Trade School	2 (6%)		
Some College/University	5 (16%)		
Diploma/Bachelor Degree	6 (19%)		
Graduate Degree	3 (9%)		
English	–	–	–
English	35 (95%)		
Other	2 (5%)		
Morbidity	3.54	2.18	0–9
**Treatment condition**	–	–	–
AR	18 (49%)		
No-AR	19 (51%)		
**Psychological variables** ^**b**^			
Helplessness	1.03	1.00	0–3
Perceived control over health	5.86	1.89	2–10
Perceived control over fitness	6.05	1.89	2–10
Perceived control over life in general	6.65	1.83	3–10

Variables were coded categorically for sex (1 = male, 2 = female).

a Pre-treatment measures.

b Post-treatment measures. Five participants did not disclose their education completed.

The study procedure spanned seven months with three sequential steps. Prior to Step 1, health records were collected from the hospital (e.g., MMSE scores) and participants were randomly assigned to either the AR or No-AR condition. Forty-four participants were eligible for the study; however, seven chose not to participate. Step 1 entailed both groups completing a Time 1 (T1) survey comprising demographic information and control-related variables. During Step 2 (1-week later), participants in the treatment condition (*n* = 18) viewed the AR video and subsequently filled out a Time 2 (T2) post-treatment survey. Participants in the No-AR condition (*n* = 19) simply filled out the survey. At each step, participants received $10 for participating.

### Measures

#### Independent variables

AR comprised an 18-min video with the intention to encourage individuals to focus on the causes of their inactivity that they have control over (poor strategies, low effort) and to avoid thinking about causes they do not have control over (e.g., old age, health problems). The goal of the video was to encourage thinking in controllable ways about inactivity. The video provides a social exchange between two older adults. The first character (Jim) embraces internal and uncontrollable causes (attributions) for why he is inactive. The second character (Don) helps “retrain” Jim's causal thinking by encouraging him to use more internal and controllable causes (e.g., change his strategy by taking it slow, use a walker, initiate breaks when tired). At the end of the video, Jim effectively models Don's adaptive way of thinking and begins seeing positive changes in his motivation and activity levels. In conclusion, Jim explains why focusing on controllable causes for inactivity can be beneficial: these causes (attributions) can be modified in the future by individuals.

#### Covariates

Covariate selection was guided by past research and our preliminary analyses as outlined below. Prior research has identified age, sex and morbidity as sharing important relationships with physical activity (Caspersen et al., 2000; Penedo et al., 2004), and sex is shown to moderate the relationship between health and physical activity (Chipperfield et al., 2008). The decision to include these covariates was supported by the zero-order correlations (see preliminary analyses below). Background information pertaining to participants' sex (1 = *male*, 2 = *female*) and medical conditions were obtained from hospital records with permission obtained from the participants and the health centre. Medical conditions were measured using a sum score from participants' reports of 39 possible medical conditions (e.g., stroke, arthritis, diabetes, asthma, anemia) experienced within the last year (0 = *no*, 1 = *yes*). A score was calculated by summing the conditions; thus, higher scores denote higher ratings of multimorbidity. Participants were asked to report their age at Step 1 in the Time 1 survey. See Table 1 for a summary of the study variables.

#### Dependent variables

Participants were asked “during the last week, how often have you felt helpless?” on a 4-point scale (0 = *never*, 1 = *rarely*, 2 = *sometimes*, 3 = *often*). Domain specific (fitness, physical health) and global (life in general) measures of perceived control were assessed on a 10-point scale. Participants were asked “how much influence do you feel you have (1 = *almost no influence*, 10 = *total influence*) over your physical health/physical fitness/life in general.” These one-item measures that have been used in other perceived control studies in health domains have high face validity (e.g., Chipperfield et al., 2018).^1^ The domains of fitness and physical health were relevant since the goal of AR is to help older adults with compromised health feel more in control over their physical inactivity.

### Analysis plan

Preliminary analyses (a) tested the effectiveness of our randomization procedure using independent sample *t*-tests on pre-treatment helplessness and perceived control variables, and (b) examined zero-order correlations among all study variables. Our main analyses employed ANCOVAs to test differences between the AR and No-AR conditions for each dependent variable employing directional hypotheses. Analyses controlled for age, sex, and morbidity. Lastly, a supplemental analysis employing a Repeated Measures ANCOVA was conducted to examine change in the outcome variables from Time 1 to Time 2 for the AR and No-AR groups (controlling for age, sex, and morbidity).

## Results

First, the preliminary analyses revealed independent sample *t*-tests established the effectiveness of our random assignment to conditions. In other words, no pre-treatment differences between conditions (AR vs. No-AR) were observed for helplessness, *t*_(34)_ = 1.33, perceived control over health, *t*_(34)_ = −0.42, perceived control over fitness, *t*_(33)_ = −1.12, and perceived control over life in general, *t*_(34)_ = −0.71 (all *p*'s >0.05). Second, zero-order correlations revealed several relationships (see Table 2). Morbidity was positively associated with age (*r* = 0.33) and helplessness was negatively related to perceived control over health and fitness (*rs* = −0.47, −0.36, respectively). There were no sex differences found. The results reveal expected correlations between T1-T2 perceived control variables (*r*s range = 0.61-0.78).

**Table 2:** Zero-order correlations of main study variables.

	**1**	**2**	**3**	**4**	**5**	**6**	**7**
1. Age^a^	−						
2. Sex^a^	0.28	−					
3. Morbidity^a^	0.33^*^	< 0.01	−				
4. Helplessness^b^	0.15	−0.18	0.33	−			
5. Perceived control over health^b^	−0.13	0.13	−0.12	−0.47^**^	−		
6. Perceived control over fitness^b^	0.04	0.11	−0.16	0.36^*^	0.78^**^	−	
7. Perceived control over life in general^b^	0.17	0.30	−0.09	−0.29	0.61^**^	0.71^**^	−

Variables were coded categorically for sex (1 = male, 2 = female).

a Pre-treatment measure.

b Post-treatment measure.

* p < 0.05,

** p < 0.01 (two-tailed tests). N range 34–37 (listwise).

**Figure 1:**
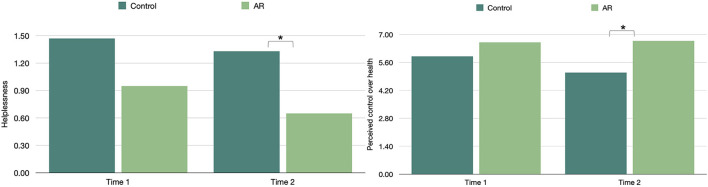
Attributional Retraining (AR) treatment differences in control-related outcomes at Time 1 and Time 2. In the sample of older adults attending a day hospital, pre- and post-treatment differences are presented between the AR (*n* = 18) and No-AR (*n* = 19) participants. Findings revealed AR (vs. no-AR) recipients reported significantly lower helplessness and greater perceived control over health at Time 2. Although the Time 1 helplessness means appear to differ in the figure, the difference was not significant.

Our hypotheses that assessed post-treatment (T2) differences between AR and No-AR participants were partially supported. An ANCOVA revealed a significant difference between the conditions on helplessness, *F*_(1,29)_ = 4.45, *p* = 0.022, $\eta_{\text{p}}^{2}$ = 0.13. Least Significant Difference (LSD) comparisons showed AR recipients (vs. No-AR) reported significantly lower helplessness post-treatment (*M*_diff_ = −0.67, *SE* = 0.32, 95% *CI* = −1.217, −0.131; Figure 1).

ANCOVAs revealed an overall significant difference between conditions at T2 in perceived control over health, *F*_(1,32)_ = 7.07, *p* = 0.006, $\eta_{\text{p}}^{2}$ = 0.18, a marginal difference in perceived control over fitness, *F*_(1,32)_ = 2.85, *p* = 0.051, $\eta_{\text{p}}^{2}$ = 0.08, but no difference in perceived control over life in general. LSD *post-hoc* comparisons indicate AR recipients (vs. No-AR) had greater perceived control over health at T2 (*M*_diff_ = 1.59, *SE* = 0.60, 95% *CI* = 0.577, 2.600; Figure 1).

A supplemental Repeated Measures ANCOVA was conducted to test for change in the outcome variables over time for the two conditions, controlling for age, sex and morbidity. For the AR group, there was a significant decline in helplessness from Time 1 to Time 2 [*F*_(1,11)_ = 4.32, *p* = 0.031]; and no difference for perceived control over health, fitness, or life in general. For the No-AR group, there was no significant change in helplessness from Time 1 to Time 2, but there was a significant decline in perceived control over health [*F*_(1,15_) = 5.47, *p* = 0.017].

## Discussion

Our randomized pilot study of older adults attending a day hospital provides evidence for AR's control-enhancing benefits. Those who received a brief AR intervention designed to encourage an adaptive way of thinking about physical inactivity reported feeling less helpless and perceiving more control over their health compared to those who did not receive AR. Although preliminary, these findings hold promise that AR treatments may be beneficial for the psychological wellbeing of older adults with compromised health.

Older adults who viewed a video on the importance of making adaptive, modifiable attributions for their inactivity subsequently reported less helplessness regardless of their sex, age, or morbidity scores. These findings align with attribution theory since attributing negative outcomes (inactivity) to unstable causes is posited to lower helplessness (Weiner, 2018). Furthermore, AR produced a positive effect on older adults' perceived control over health compared to older adults in the No-AR condition. Our results are encouraging since older adults who received the AR treatment felt more in control over their health suggesting they may be motivated to take action in engaging in healthful behaviors (e.g., increase physical activity). They also suggests individuals may have begun thinking in more controllable ways about their physical inactivity (e.g., change strategy, effort), which was the goal of AR. Supplemental analyses further suggest that AR may help to buffer against future declines in perceived control over health. There appeared to be a similar trend concerning AR (vs. No-AR) recipients' perceived control over fitness although it was not statistically significant.

Our study addresses an omission in the AR literature within the context of promoting adaptive thinking for older adults by testing AR's efficacy in a population with compromised health. This study makes a valuable contribution by suggesting that a brief intervention can augment control-related outcomes that are shown to be critical psychological variables for individuals' health, resilience in combatting adversity, and healthy behavioral patterns (Infurna et al., 2013; Robinson and Lachman, 2017). One limitation concerned our single follow-up and challenges with recruiting eligible day hospital participants that resulted in a small sample size. Thus, we acknowledge that our examination of the efficacy of an AR intervention is a preliminary step that must be replicated by studies with larger study samples and a longer follow-up period to systematically examine changes in baseline and post-treatment outcomes including physical activity. If replicated, our AR program that attempts to minimize the misconception that physical activity is beyond them, has the potential to benefit older adults who are inactive. Such programs can offer a brief, scalable intervention that can be implemented across community and clinical settings by clinicians, social-workers, and researchers interested in improving the psychological wellbeing of older adults with health challenges. Helping them to avoid the psychological state of helplessness and being out-of-control and, at the same time, promoting their physical activity and mobility also has the potential to enhance independence and overall quality of life.

### Recommendations

Our study conveys the importance of shifting attributions about outcomes—where subtle changes can have a big impact on health and motivation. Attribution-based treatments have been replicated and effective in achievement contexts (e.g., see Perry et al., 2014) but their use in health and clinical settings is still underused considering the detrimental impact uncontrollable attributions have for older adults (Parker et al., 2019). We recommend that practitioners and educators of all ages consider the importance of reframing detrimental thinking about the origins of inactivity in ways that motivate people to be active. Especially important is the need to encourage more adaptive ways of thinking among those who may feel helpless.

## Data availability statement

The raw data supporting the conclusions of this article will be made available by the authors, without undue reservation.

## Ethics statement

The studies involving human participants were reviewed and approved by University of Manitoba Psychology/Sociology Research Ethics Board (#P2019:052 HS22789). The patients/participants provided their written informed consent to participate in this study. Ethics approval was also received from the Riverview Health Centre.

## Author contributions

JC, JH, RP, SH, LC, and PP conceptualized the project and defined the methodology. PP, MK, and LC ran the data collection. PP and JC conducted the analyses and wrote the first manuscript draft. All authors agree to be accountable for the content of the work, contributed sufficiently to the manuscript to justify authorship, took part in interpreting and writing the results, and editing the final version of the manuscript.

## Funding

This work was supported by the Social Sciences and Humanities Research Council of Canada under JC Grant [435-2016-0970].

## Conflict of interest

The authors declare that the research was conducted in the absence of any commercial or financial relationships that could be construed as a potential conflict of interest.

## Publisher's note

All claims expressed in this article are solely those of the authors and do not necessarily represent those of their affiliated organizations, or those of the publisher, the editors and the reviewers. Any product that may be evaluated in this article, or claim that may be made by its manufacturer, is not guaranteed or endorsed by the publisher.

## Footnotes

^1^Analyses assessing the distributions of the dependent variable residuals for each treatment group indicated distributions roughly approximated normality (e.g., Shapiro Wilk statistic range = 0.913 to 0.977, all *p*'s > 0.05).
